# An Electrical Equivalent Model of an Electromembrane Stack with Fouling Under Pulsed Operation

**DOI:** 10.3390/membranes16010042

**Published:** 2026-01-16

**Authors:** Pablo Yáñez, Hector Ramirez, Alvaro Gonzalez-Vogel

**Affiliations:** 1 Department of Electronic Engineering, Universidad Tecnica Federico Santa Maria, Valparaiso 2390123, Chile; hector.ramireze@usm.cl; 2 Anturi SpA, Av. Andalue 2451A, San Pedro de la Paz 4130000, Chile; alvaro.mgv@anturi.cl

**Keywords:** electromembrane, modeling equivalent circuit, desalination, fouling, pulsed operation

## Abstract

This study introduces a novel hybrid model for an electromembrane stack, unifying an equivalent electrical circuit model incorporating specific resistance ($R_{M},R_{s}$) and capacitance ($C_{gs},C_{dl}$) parameters with an empirical fouling model in a single framework. The model simplifies the traditional approach by serially connecting *N* (N=10) ion exchange membranes (anionic PC-SA and cationic PC-SK) and is validated using NaCl and $Na_{2}SO_{4}$ solutions in comparison with laboratory tests using various voltage signals, including direct current and electrically pulsed reversal operations at frequencies of 2000 and 4000 Hz. The model specifically accounts for the chemical stratification of the cell unit into bulk solution, diffusion, and Stern layers. We also included a calibration method using correction factors ($\alpha_{i}$) to fine-tune the electrical current signals induced by voltage stimulation. The empirical component of the model uses experimental data to simulate membrane fouling, ensuring consistency with laboratory-scale desalination processes performed under pulsed reversal operations and achieving a prediction error of less than 10%. In addition, a comparative analysis was used to assess the increase in electrical resistance due to fouling. By integrating electronic and empirical electrochemical data, this hybrid model opens the way to the construction of simple, practical, and reliable models that complement theoretical approaches, signifying an advance for a variety of electromembrane-based technologies.

## 1. Introduction

Modeling is a tool that has been used for decades for explaining phenomena and optimizing processes. In the realm of fundamental studies, mathematical models are employed to explain physicochemical phenomena. Conversely, engineering models, which are predicated on operational data, aim to predict the behavior of various processes [1].

Electromembrane technologies, including Electrodialysis (ED), Bipolar Membrane Electrodialysis, Electrodeionization, Selectodialysis, Electrodialysis Reversal, Metathesis ED, Reverse Electrodialysis, Capacitive Deionization, Electro-Electrodialysis, Shock Electrodialysis, Electrodialytic Crystallization, Fuel cells, PEM and AEM electrolysis for hydrogen production, among others, play an important role in various industrial applications due to their efficiency in manipulating ionic components in aqueous solutions. ED, a particularly versatile technique, is probably the most common one, which primarily facilitates the removal of ions from solutions [2]. This technology can be adapted for a range of applications depending on the configuration of membranes, electrodes, feed order, and fillers. Such adaptations make ED suitable for the recovery of chemicals, energy generation, and the production of ultrapure water, thereby serving diverse industries.

The ED system consists of multiple components such as water pumps, sensors, pipes, power sources, and membrane stacks, each containing a series of cells formed by ion exchange membranes and spacers. These cells are organized in a configuration that positions hundreds between electrodes, an anode and cathode. Unlike filtration methods, ED operates by having the solution flow tangentially across the membranes, separating ions through migration driven by an electric field, established by a potential difference applied in the end electrodes via a direct current. Moreover, the versatility of the ED infrastructure supports alternative operations like Reverse Electrodialysis (RED) to harvest energy from saline gradients [3,4,5], which use the same structural components but different operating principles.

Several studies have modeled the ED process, highlighting some aspects such as different electrical models of ED stacks that focus on specific electrochemical behaviors of their layers [6], optimal calculations for limit current densities [7,8], and energy efficiency [9].

Furthermore, some researchers examine the influence of input effluent composition [10,11], conduct experiments for parameter identification [12], and investigate systems using only a single ion exchange membrane [1,13].

The mathematical modeling of electromembrane stacks is notably challenging due to the multitude of factors influencing their performance. Each of these elements can significantly alter the behavior and efficiency of the process, necessitating detailed and multiphysical modeling approaches to capture the nuances of electromembrane operations in an effective way. These factors include mainly hydrodynamics; electrochemical potential; applied electric field strength; utilization of turbulence promoters; characteristics of ion exchange membranes, temperature, pH, stack geometry, fouling, and biofouling; and scaling of membranes.

**Figure 1:**
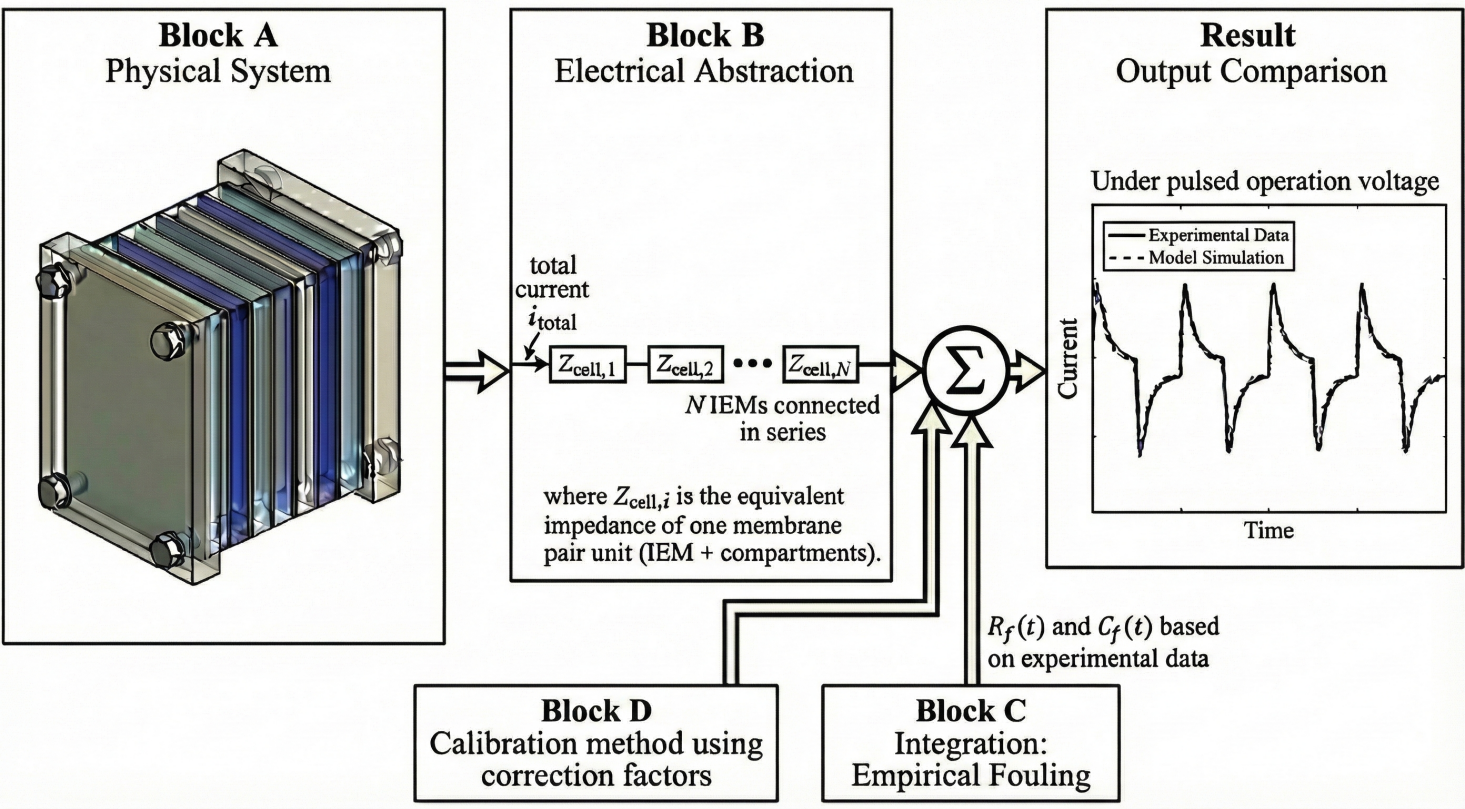
Schematic representation of the conceptual framework.

Figure 1 presents a schematic diagram of the conceptual framework, illustrating the transition from the physical system to the proposed integrated hybrid model.

Despite these advancements, the mathematical modeling of electromembrane stacks remains notably challenging due to complex factors such as hydrodynamics, stack geometry, and especially membrane fouling, biofouling, and scaling. Fouling is a ubiquitous challenge that increases energy consumption and maintenance requirements [14,15,16,17,18,19]. Although innovative solutions like pulsed Electrodialysis Reversal (pEDR) or Pulsed Electric Fields (PEF) [20,21,22] can mitigate these effects, research on the pulsed operation of the complete stack is remarkably limited, as most studies focus strictly on single-membrane systems. In this study, we developed a hybrid model of electromembrane stacks. This work specifically expands upon the equivalent electrical model of a single ion exchange membrane (IEM) [1]. It hypothesizes that by serially connecting this equivalent electrical system *N* times, accounting for variable membrane properties, a more accurate and realistic model can be achieved, this arrangement of *N* assumes that all IEMs possess uniform properties. Finally, the model was expanded to cover fouling growth based on laboratory experiments to ensure a simple and practical approach for the simulation of electromembrane processes.

## 2. Materials and Methods

A proposed equivalent electrical model was designed for electromembrane stacks. A cell structure (a repetitive unit in the stack) was stratified into several layers, which include a bulk solution, a diffusion layer, a Stern layer, and ion exchange membranes. If modeled, the electromembrane cell (basic unit) accounts for various components such as membrane resistance ($R_{M}$), geometric capacitance ($C_{M}$), diffusion-layer resistance ($R_{s}$), and geometric capacitances ($C_{gs}$ and $C_{dl}$).

To extend the approach to more complex setups (stacks), the model was adapted to include multiple ion exchange membranes. Equation (1), detailing the equivalent impedance of an electromembrane stack with *N* membranes, is provided to support this extension. Additionally, the model addresses fouling by integrating components like fouling resistance ($R_{f}$) and capacitance ($C_{f}$), whose values are empirically determined. These electrical components are incorporated because fouling is modeled as a discrete layer that accumulates on the membrane surface. Further details on the chemicals and membranes (Table 1) utilized in the study are provided, along with comprehensive descriptions of the desalination process under pulsed conditions, which previously illustrates a reduction in the occurrence of fouling.

### 2.1. Basal Components of the Model

The electromembrane stack is composed of multiple layers, each characterized by distinct electrical properties. Central to this configuration is the ion exchange membrane, characterized by its resistance ($R_{M}$), which is influenced by the chemical nature and thickness [3].

**Figure 2:**
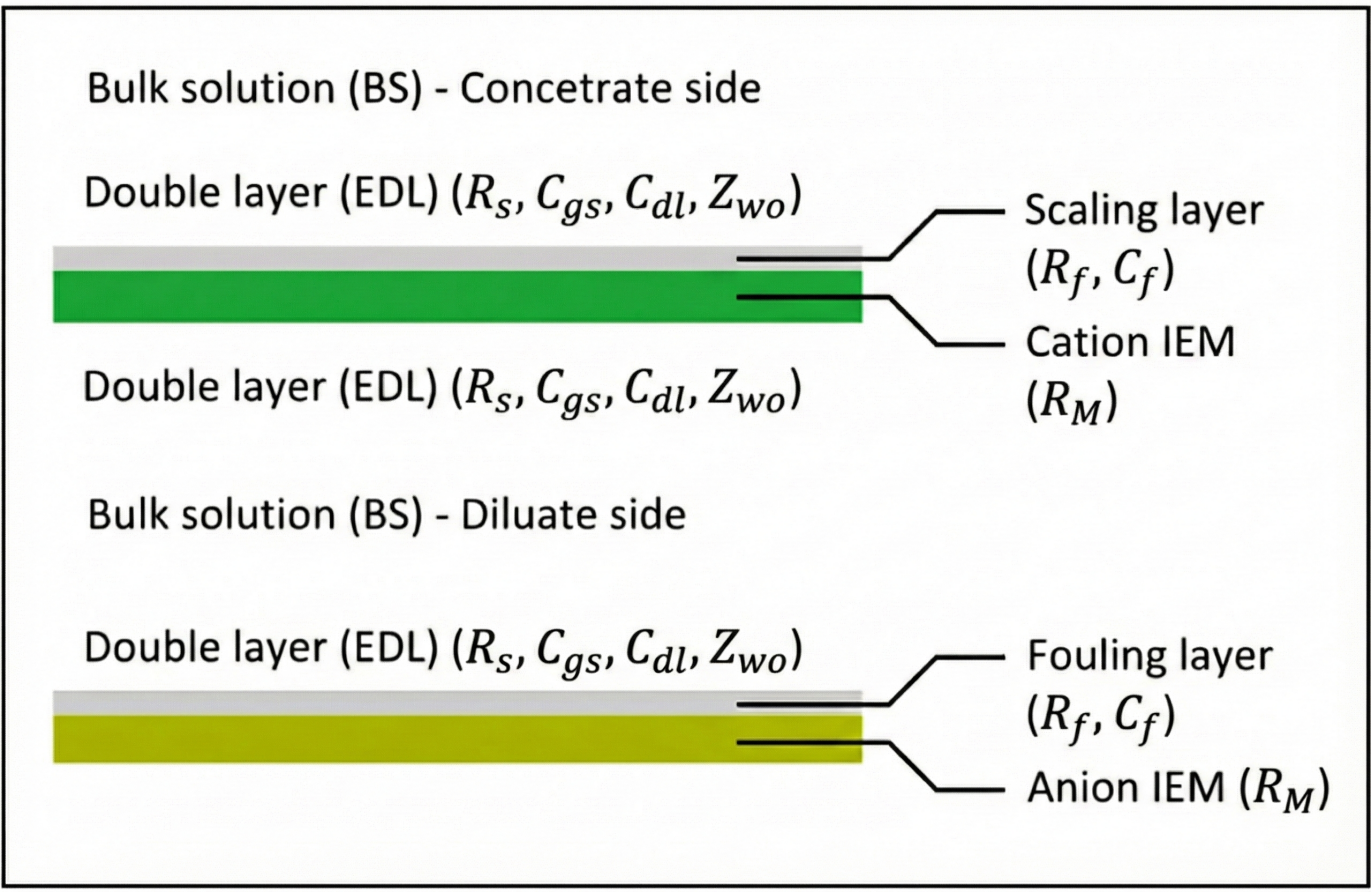
Schematic representation of the electrodialysis cell layers with the integrated electrical components used to characterize resistance and capacitance across the membrane system and fouling layers.

For the purposes of this study, it is assumed that the IEM is fully covered by the fluid being treated throughout its entire effective area. The cell, the basic and repetitive electromembrane unit, typically consists of both anion and cation exchange membranes in ED (Figure 2). This cell is stratified into several distinct layers as mentioned before: a bulk solution (BS), a diffusion layer (DL), a Stern layer (SL), and the IEM.

It is important to note that the electric double layer (EDL) comprises both the DL and SL. The distribution of these layers has a specific order, and its extension depends on how many membranes the electromembrane stack has, from electrode to electrode, based on the required flow to be treated.

Within the IEM layer, the geometric capacitance ($C_{M}$) is connected in parallel to $R_{M}$ but is effectively considered negligible (zero farads) because the charge within the IEM layer remains fixed, thus $C_{M}$ does not significantly affect the overall model beyond the contribution of $R_{M}$.

The diffusion layer forms as ions migrate through the membranes from the bulk solution, under an electric field. This layer is limited at higher current densities, where no further ions are available on the surface of the membranes after pulling them all, known as the Stern layer. Typically, the diffusion layer has a thickness in the order of micrometers. The combined resistance of the diffusion and Stern layers is referred as $R_{s}$, and their geometric capacitance, represented as $C_{gs}$ [1], accounts for the charge differences and the width of the double layer, also in the order of micrometers.

Additionally, the diffusion layer with the bulk solution, which is rich in fresh ions are modeled using the geometric capacitance $C_{dl}$, considering the charge differences and the width of the Stern layer. Complementary, the Warburg Impedance of finite length, represented by $Z_{wo}$, is used to model the interaction between layers, and assumes a Donnan equilibrium, reflecting the influence of both the Stern and diffusion layer [1,23]. Moreover, given the frequency range used in this study (In the order of kilohertz at most), the effect of $Z_{wo}$ is assumed to be purely resistive.

#### 2.1.1. Addition of an Indefinite Number of Membranes to the Equivalent Electrical System

**Figure 3:**
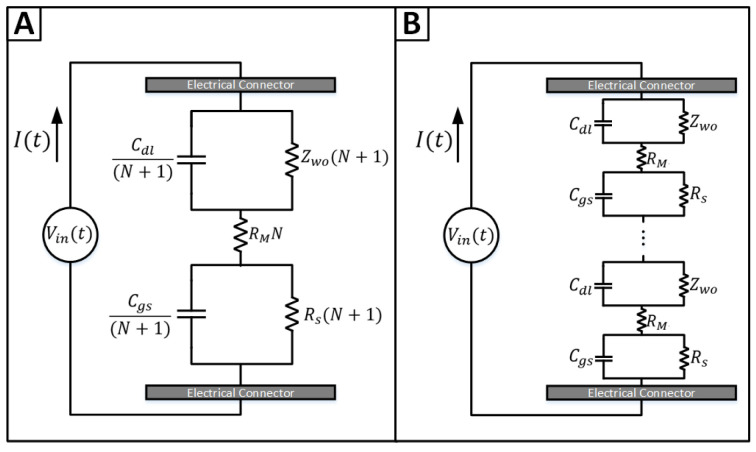
$Z_{StackNoFouling}$: Basal construction of the electromembrane stack model using an electronic approach. (**A**) Equivalent electrical circuit model of the stack. (**B**) Generalization of the equivalent model when integrating different membranes in series.

Electronically, each layer within the electromembrane stack is composed of a resistance in parallel with a capacitance, connected in series to the corresponding components of adjacent layers, except for the membrane resistance, which lacks a parallel capacitance. The characteristics of these components are specific to the layer in which they are located. For configurations involving *N* ion exchange membranes, the electrical model was derived by integrating models referenced in previous studies [1], with the considerations outlined above and arranging them in series (Figure 3A). This arrangement assumes that all membranes possess uniform properties. Should any membrane, or a group thereof, exhibit distinct physical or chemical properties, it would result in varied electrical behaviors and necessitate additional components in the model.

**Figure 4:**
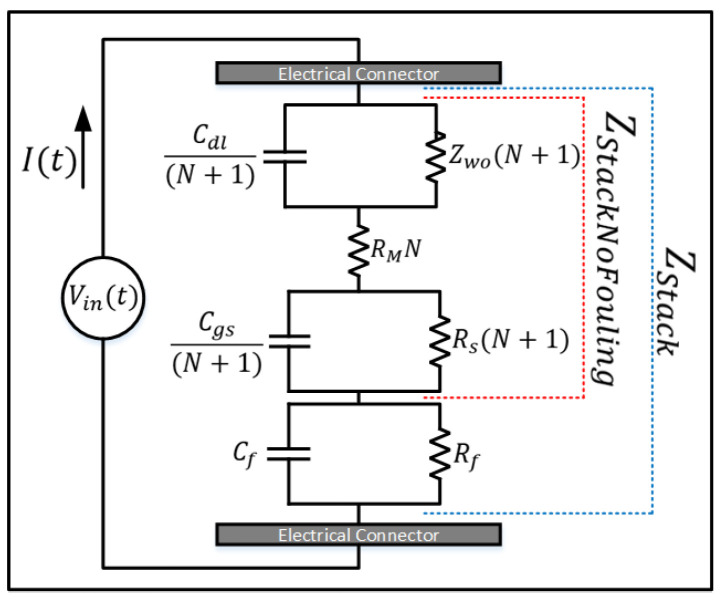
$Z_{Stack}$: Representation of an equivalent electrical circuit model of an electromembrane stack, with the fouling layer incorporated into the overall model. The model is structured to simulate the electrical behavior of the stack under different operational conditions, including the presence of fouling, which is a common issue in longterm membrane operations.

The multiplicative factor of *N* is applied by connecting all the membrane subcircuits in a series (Figure 3B). By reducing the number of components and obtaining the equivalent circuit, the *N* factor is multiplied to the resistances and divided by the capacitances. By applying the Laplace transform [24] to the equivalent circuits shown in Figure 4, we obtain

(1) $$Z_{Stack}={\left(\frac{1}{R_{s}\left(N+1\right)}+\frac{C_{gs}}{\left(N+1\right)}s\right)}^{-1}+{\left(\frac{1}{Z_{wo}\left(N+1\right)}+\frac{C_{dl}}{\left(N+1\right)}s\right)}^{-1}+{\left(\frac{1}{R_{f}}+C_{f}s\right)}^{-1}+R_{M}N.$$

which is derived as a summation of four components.These consist of two terms representing the EDL (comprising $R_{s}$, $C_{gs}$, $Z_{wo}$, and $C_{dl}$), one term representing the IEM ($R_{M}$), and one term representing the fouling layer ($C_{f}$ and $R_{f}$). By excluding the component representing the fouling layer,

(2) $$Z_{StackNoFouling}={\left(\frac{1}{R_{s}\left(N+1\right)}+\frac{C_{gs}}{\left(N+1\right)}s\right)}^{-1}+{\left(\frac{1}{Z_{wo}\left(N+1\right)}+\frac{C_{dl}}{\left(N+1\right)}s\right)}^{-1}+R_{M}N.$$

is obtained. The equivalent impedance of the electromembrane stack with *N* membranes is denoted as $Z_{Stack}$. An “N + 1” factor is employed because each additional membrane introduces two identical circuits on both the left and right sides of an ion exchange membrane [1]. However, for $R_{M}$ specifically, it is multiplied only by N, as it directly correlates to the number of membranes. $Z_{StackNoFouling}$ represents the impedance of the stack without the components that account for fouling (Figure 3), while $Z_{Stack}$, which includes all components, is shown in Figure 4.

#### 2.1.2. Incorporation of Fouling Electric-Equivalent Components

Fouling and scaling in electromembrane systems occur primarily due to the presence of organics (fouling) and inorganics (scaling), leading to periodic deposition and formation of layers on the membrane surfaces (Figure 2). These layers exhibit resistive and capacitive behaviors. To model them, fouling and scaling are considered equivalent and collectively referred as “fouling” from this point onward, as their impact on voltage and electric current within the electromembrane stack is similar [25]. This approach is justified by the phenomenon of combined fouling, where organic and inorganic foulants interact to form a synergistic composite layer rather than distinct strata [26,27]. Furthermore, this composite accumulation acts as a high-resistance dielectric barrier [26] capable of generating opposing electric fields [28], thereby exhibiting the simultaneous resistive and capacitive behaviors characteristic of the proposed $R_{f}$ and $C_{f}$ sub-circuit.

Fouling is simplified as a layer characterized by a resistance, $R_{f}$, and a capacitance, $C_{f}$, connected in parallel, similar to other described layers. The values of $R_{f}$ and $C_{f}$ primarily depend on the feeding time of the water sample containing foulants. As the electromembrane system runs, fouling increases over time, and the rates at which $R_{f}$ and $C_{f}$ change as well when varying the electric current. As mentioned in the introduction, pEDR or PEF operation modes could decrease the fouling occurrence in electromembrane processes by disturbing and releasing the accumulated dirt [20].

The values of $R_{f}$ and $C_{f}$ are empirically determined based on the fouling observed in an electrodialysis system operating under pEDR or PEF modes with a sample containing foulants. Over a specific period of operation, these components provide an approximation of the growing resistance due to fouling within the system under the given operational conditions.

### 2.2. Chemicals and Membranes

NaCl and Na_2_SO_4_ (analytical grade) were obtained from Merck Chemicals, Darmstadt, Germany. Standard desalination membranes PC-SA (anionic exchange) and PC-SK (cationic exchange) were obtained from PCCell GmbH, Marpingen, Germany. PC-SA and PC-SK have an electrical resistance of 1.8 and 2.5 $\frac{\Omega }{{\mathrm{cm}}^{2}}$ respectively.

### 2.3. Electromembrane System

The employed electromembrane system includes a power supply, pumps, automatic valves, and an electromembrane stack (ED64, PCCell GmbH, Germany). Five pairs of IEMs provided (N = 10) a total surface area of $320\;{\mathrm{cm}}^{2}$, along with an individual membrane area of $64\;{\mathrm{cm}}^{2}$. The system was used as electrodialysis for desalting a solution of NaCl, with a linear velocity of $6\;\frac{L}{\mathrm{cm}}$. The electrode compartment had a rising solution of 0.25 M Na_2_SO_4_, along with a flow rate of $0.4\;\frac{L}{\min}$.

### 2.4. Pulsed Operation

Coupling electromembrane stack of an Asymmetric Bipolar Switch (ABS), Anturi SpA, Concepcion, Chile, allows the application of reverse polarity pulses at different conditions, varying frequencies, pulse widths and intensities [29].

In this study, a frequency of 2000 Hz and 4000 Hz, an amplitude of 23 Vdc, and pulse widths of 10 µs and 50 µs, were used to validate the model.

## 3. Results and Discussion

The values of the components associated with the electrical equivalent circuit of the electrodialysis process are presented. Subsequently, a method to calibrate these values is introduced, and finally, the components representing fouling are added, thus obtaining the complete and calibrated representation of the electrodialysis system.

### 3.1. Theoretical Electrical Component Values

Initially, the component values associated with the electrical equivalent circuit were computed and analyzed. The precision of the equivalent circuit in the electrical model was directly reflected in the electrical current readings. These readings were key to evaluate the effectiveness of the determined values and distribution of components within the modeled circuit.

The results obtained from the model provided us insights into the steady-state value of the electric current, the magnitude of electric current peaks, and the settling time required for the transient electric current to reach a steady state when the voltage source is switched during pulsed operation.

Thus, an ED stack of 5 pairs of ion exchange membranes (N = 10) of $64\;{\mathrm{cm}}^{2}$ of effective area, and 23 Vdc was analyzed. A voltage drop of 3 V was used for the electrodes [29], and the membrane resistance was $R_{M}=107.14\;\Omega {\mathrm{cm}}^{2}$, obtained directly from the peak value of the electric current that divides the continuous supply voltage [3], the capacitance associated with the ion exchange membrane was considered to have a value of zero (given its low charge difference and to facilitate calculations).

On the other hand, $R_{s}=2.6021\;\Omega {\mathrm{cm}}^{2}$ [1] was obtained assuming that the average electric current is at least 80% of the limiting current density, the temperature of the process was kept constant at 20 °C (293.15 °K), with a diffusion coefficient D1 equal to D2 [1] and effective transport number t1 and t2 had a value of 0.5 [1], with an initial concentration of 0.25 M NaCl [20] and an empirical value of limiting current density of $15.625\;\frac{A}{{\mathrm{cm}}^{2}}$ [20]. Additionally, $C_{gs}=5\;\mu \frac{F}{{\mathrm{cm}}^{2}}$ [1] was obtained considering a width of the DBL layer in the order of 100 µm and a relative permittivity of water, in the range of 80 to 100. $Z_{wo}=9.3772\;\Omega {\mathrm{cm}}^{2}$ [1], was obtained under the same considerations as $R_{s}$, but also considering that jω→0, since the amplitude in its imaginary component was negligible as frequencies in the order of MHz were not used, so $Z_{wo}$ was analyzed as a resistor.

Finally, $C_{dl}=43.7\;m\frac{F}{{\mathrm{cm}}^{2}}$ [1] was calculated in the EDL layer under the same conditions as above. Then by considering 10 IEMs and a total effective area of $64\;{\mathrm{cm}}^{2}$, we obtained the final values, shown in Table 1.

In Table 1, The Density Value column represents the resistance or capacitance depending on the effective area of an IEM, and the Total column represents the value considering an effective area of 64 cm^2^; of *N* = 10 IEMs. the values of the components that make up the $Z_{StackNoFouling}$ are obtained, whth this, the electric current can be observed given a constant voltage, pEDR, or PEF signal.

When simulating the parameters obtained from Table 1 for the $Z_{stackNoFouling}$ model, the shape of the electrical current signal matches those reported in T11 [20]. However, these signals exhibit errors around a 10% concerning the peak current magnitude ($i_{Max}$) and the magnitude just before switching to negative voltage ($i_{Com}$) during pEDR operation with $V_{in}=23\;V$, 4000 Hz and pulse width of 10 μs.

### 3.2. Electrical Components Calibration

To adjust the simulation constants the following parametrization was used.

(3) $$R_{M-calib.}=R_{M}\alpha_{1}$$

(4) $$C_{dl-calib.}=C_{dl}\alpha_{2}$$

(5) $$C_{gs-calib.}=C_{gs}\alpha_{3}$$

(6) $$R_{s-calib.}=R_{s}\alpha_{4}$$

(7) $$Z_{wo-calib.}=Z_{wo}\alpha_{5}$$

with $\alpha_{i}$, i=1,2,3,4,5 being the parameters used to adjust the electrical parameters. The laboratory data was obtained from: Peak electrical current value $i_{Max}$ and electrical current value at the instant in which the voltage source switches from positive to negative values ($i_{Com}$) in pEDR operation reported in T11 [20], these parameters were selected based on the assumption that the electric current reaches a steady state before each subsequent voltage pulse.

**Figure 5:**
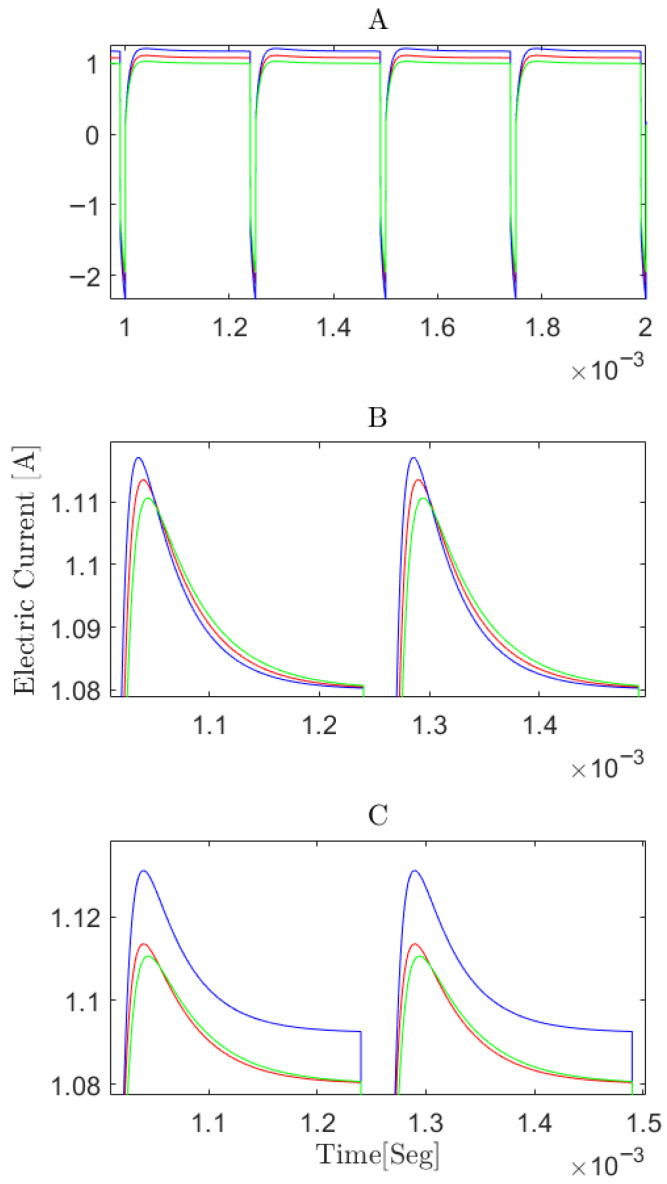
Simulation of T11 experiment [20] with $V_{in}=23\;V$, 4000 Hz, pulse width of 10 μs: (**A**) changing $R_{M}$ ion exchange membrane resistance, with unchanged $R_{M}$ (red line), 90% of $R_{M}$ (blue line), and 110% of $R_{M}$ (green line). (**B**) changing the capacitances, with $C_{dl}$ and $C_{gs}$ unchanged (red line), $C_{dl}$ and $C_{gs}$ at 90% (blue line), and $C_{dl}$ and $C_{gs}$ at 110% (green line). (**C**) changing resistances $Z_{wo}$ and $R_{s}$, with unchanged (red line), $Z_{wo}$ and $R_{s}$ at 90% (blue line) and with $Z_{wo}$ and $R_{s}$ at 110% (green line).

Accordingly, $\alpha_{1}$ was varied until the simulated electric current peak value ($i_{SimMax}$) and the simulated electric current steady-state value ($i_{SimCom}$) approach to $i_{Max}$ and $i_{Com}$, respectively, and then $\alpha_{4}$ and $\alpha_{5}$ were varied for micro tuning purposes of the above. Subsequently, $\alpha_{2}$ and $\alpha_{3}$ were varied only to adjust the settling time of the electric current in such a way that without a significant change of $i_{SimMax}$, the value of $i_{SimCom}$ was corrected. When the value of $R_{M}$ (Figure 5a) changed, only the amplitude of the electric current signal was affected. This is because $R_{M}$ does not have a parallel capacitance, thus influencing only the magnitude of the current signal. As shown in Figure 5a, even small variations in this component can result in significant changes in the peak and settling value of the electric current reached.

Figure 5b illustrates that when varying the values of $C_{dl}$ and $C_{gs}$, the settling time of the electric current is primarily impacted. This suggests a straightforward method of calibrating the settling time through adjustments of these components. While $Z_{wo}$ and $R_{s}$ (Figure 5c) also undergo changes in magnitude similar to $R_{M}$, their total contributions are at least 10 times smaller than the capacitances based on the proportions of the values obtained (as shown in Table 2). Membrane resistance ($R_{M}$) exerts the dominant influence on signal amplitude, whereas $Z_{wo}$ and $R_{s}$ exhibit the lowest sensitivity [1].

The correction factor $\alpha_{1}$ greatly varies the peak and steady-state magnitude of the electric current, $\alpha_{2}$ together with $\alpha_{3}$ only the settling time of the electric current and $\alpha_{4}$ together with $\alpha_{5}$ the settling time and peak and steady-state magnitude of the electric current but in a small proportion.

The calibration values α must be in the following ranges:

(8) $$1-\frac{1}{N}<\alpha_{1}<1+\frac{1}{N}$$

(9) $$1-\frac{1}{N+1}<\alpha_{\left\{2,3,4,5\right\}}<1+\frac{1}{N+1}$$

Equations (8) and (9) must be satisfied in such a way that the model continues to represent *N* IEMs; otherwise, the equivalent electrical model does not represent an ED with *N* IEMs. In other words, the calibration can deviate by a maximum of 1 IEMs in relation to the total number of IEMs. For example, if $\alpha_{1}=1.21$ with *N* = 10 IEMs, it means that the calibration does not match. In this case, $\alpha_{1}=1.21$ would imply that the electrodialysis system has 12.1 IEMs, which is not consistent with the intended calibration.

### 3.3. Model Validation Under Pulsed Operation

In the Equivalent model of the ED obtained (according to Table 3) using

(10) $$Z_{StackNoFouling}\left(t\to \infty \right)=R_{s}\left(N+1\right)+Z_{wo}\left(N+1\right)+R_{M}N$$

that represents the resistance of ED in steady state, we obtain $Z_{StackNoFouling}\left(t\to \infty \right)=18.7993\;\Omega$ which implies a steady state current of I(t→∞)=1.06A. Electrical settling time occurs within milliseconds, whereas $R_{f}$ and $C_{f}$ model long-term time transient [30].

**Figure 6:**
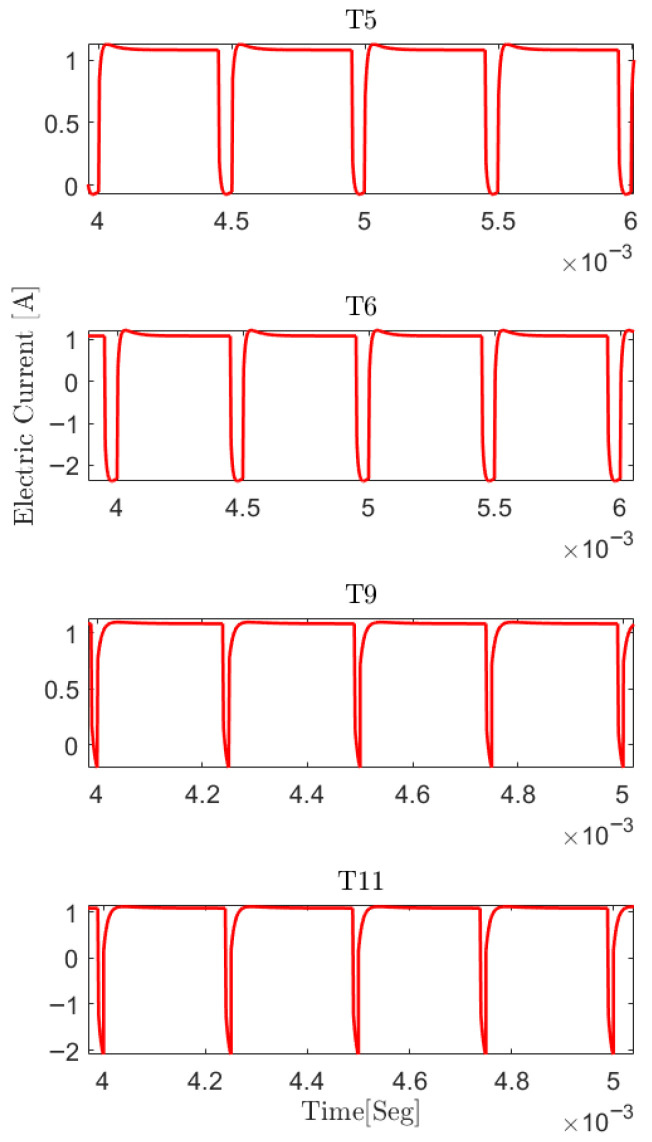
Pulsed operation pEDR simulated. The response in electric current given a voltage of amplitude 23 Volt and frequencies of 2000 Hz and pulse width of 50 μs (T5 and T6) and 4000 Hz and pulse width of 10 μs (T9 and T11).

In order to fully validate the hybrid model, different pEDR operations (Table 4) were simulated with the proposed and calibrated model (Figure 6) and compared with experimental results.

**Figure 7:**
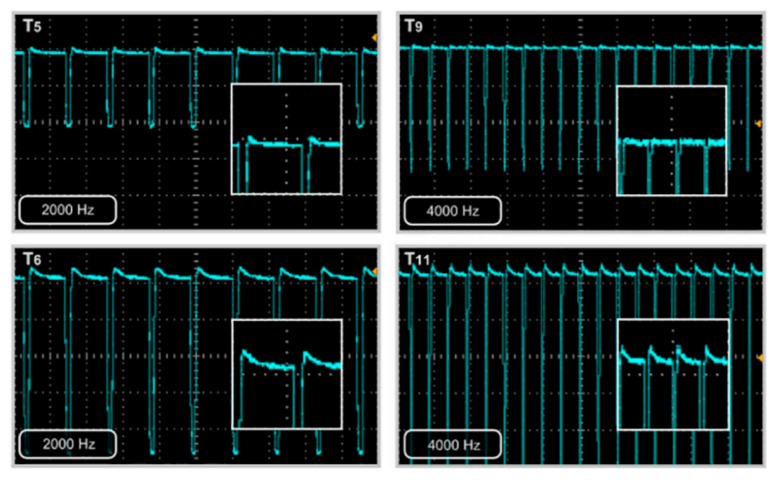
Pulsed operation pEDR over an ED described in Section 2.4 [20], The response in electric current given a voltage of amplitude 23 Volt and frequencies of 2000 Hz (T5 and T6) and 4000 Hz (T9 and T11).

The shapes of the electrical current signal obtained in simulations were similar to those obtained in the real experiments [20] (see Figure 7).

The values of interest are iSimMax and iSimCom, obtaining an error of less than 10% with respect to the empirical results (iMax and iCom), satisfying Equations (8) and (9).

To further emphasize the significance of the proposed model, it is essential to contextualize its application within the current state of the art of electromembrane processes. Membrane fouling is a dynamic phenomenon governed by complex interactions between feed characteristics, membrane properties, and electrochemical conditions. As detailed in Table 5, various case studies highlight how different types of fouling—organic, inorganic, and biological—directly impact critical performance metrics such as Specific Energy Consumption (SEC), membrane resistance, and current efficiency. By providing a simplified yet accurate electrical representation of these phenomena, the hybrid model presented in this study offers a practical tool for addressing these pervasive operational challenges.

Long-term time operational stability is fundamentally compromised by irreversible organic poisoning [26] and biofilm growth, which degrade membrane conductivity and significantly increase specific energy consumption [36]. However, these deleterious effects can be managed through optimized pulsed electric operations (pEDR and PEF) and enhanced hydrodynamic cleaning protocols, extending the functional lifespan of the membrane stack.

## 4. Conclusions

An equivalent electrical model for electromembrane stacks was proposed, integrating an equivalent electrical circuit incorporating membrane resistance ($R_{M}$), geometric capacitances ($C_{gs},C_{dl}$), and Warburg impedance ($Z_{wo}$) with an empirical fouling representation. By structuring the system as a series of 10 ion exchange membranes (5 cell pairs), the model simplifies conventional approaches while maintaining consistency with experimental observations. Validation against laboratory data using NaCl and $Na_{2}SO_{4}$ solutions confirms its ability to simulate electrical responses under various voltage conditions, including direct current and pulsed reversal operations at frequencies of 2000 and 4000 Hz. Major findings demonstrate that the model accurately predicts a steady-state resistance of 18.7993 Ω and a current of 1.06 A for the validated stack. In addition, the inclusion of a calibration method based on correction factors ($\alpha_{i}$) refines the current predictions to achieve an error margin of less than 10% compared to empirical data, while the empirical component effectively captures fouling-induced resistance ($R_{f}$) and capacitance ($C_{f}$) changes. By combining theoretical modeling with empirical electrochemical data, this approach offers a practical framework for analyzing electromembrane processes under complex operational conditions. The validity of these findings is established for an applied voltage of 23 Vdc, a frequency range of 2000–4000 Hz, and 0.25 M NaCl solutions.

## Figures and Tables

**Table 1:** Values of electric components.

Electric Component	Density Value	Total
$R_{M}$	$107.14\;\Omega {\mathrm{cm}}^{2}$	16.74Ω
$C_{dl}$	$43.7\;{\mathrm{mF}/\mathrm{cm}}^{2}$	254.254mF
$C_{gs}$	$5\;\mu {F/\mathrm{cm}}^{2}$	29.09μF
$R_{s}$	$2.6021\;\Omega {\mathrm{cm}}^{2}$	0.44723Ω
$Z_{wo}$	$9.3772\;\Omega {\mathrm{cm}}^{2}$	1.6117Ω

**Table 2:** Values of electric components calibrated.

Component	Density Value	Total	Gain
$R_{M-calib.}$	$105.946\;\Omega {\mathrm{cm}}^{2}$	16.554Ω	$\alpha_{1}=0.99$
$C_{dl-calib.}$	$43.7\;{\mathrm{mF}/\mathrm{cm}}^{2}$	254.254mF	$\alpha_{2}=1$
$C_{gs-calib.}$	$5\;\mu {F/\mathrm{cm}}^{2}$	29.09μF	$\alpha_{3}=1$
$R_{s-calib.}$	$2.839\;\Omega {\mathrm{cm}}^{2}$	0.488Ω	$\alpha_{4}=1.09$
$Z_{wo-calib.}$	$10.223\;\Omega {\mathrm{cm}}^{2}$	1.757Ω	$\alpha_{5}=1.09$

**Table 3:** The “Pulse width” is the duration in microseconds of the reverse polarity pulse, “pEDR amplitude” is the amplitude of the voltage when it is in the “Pulse width” period.

Test	Voltage Supply Parameters		
	Pulse Width [μs]	Frequency [Hz]	pEDR Amplitude
T5	50	2000	0
T6	50	2000	1
T9	10	4000	0.2
T11	10	4000	2

**Table 4:** Electric current obtained from the simulations and compared with empirical results.

Test	Error [%]		Simulated Current [A]		Empirical Current [A]	
	$i_{\mathrm{SimMax}}$ and $i_{\mathrm{Max}}$	$i_{\mathrm{SimCom}}$ and $i_{\mathrm{Com}}$	$i_{\mathrm{SimMax}}$	$i_{\mathrm{SimCom}}$	$i_{\mathrm{Max}}$	$i_{\mathrm{Com}}$
T5	5.8	8.4	1.20	1.07	1.13	0.98
T6	6.4	6.0	1.24	1.17	1.32	1.1
T9	8.4	9.0	1.19	1.10	1.09	1
T11	4.9	2.6	1.23	1.17	1.29	1.2

**Table 5:** State-of-the-Art Analysis of Fouling in Electromembrane Processes: Case Studies and Performance Metrics.

Fouling Type	Case Study Context	Specific Foulants	Consequences and Metrics	Ref.
Organic Fouling	NaCl Desalination (ED)	Sodium Humate	Increased resistance and reduced limiting current density.	[28]
Organic Fouling	Reverse Electrodialysis (RED)	Humic Acid	Severe power density reduction; mitigation via DC field > 8 V.	[31]
Organic Fouling	Model Wastewater	Surfactants (SDS, CPC)	Resistance rise; lower fouling in high ionic strength due to EDL shielding.	[32]
Organic/Mineral	Tofu Whey Recovery	Isoflavones, Proteins, $Mg^{2+}$	AEM conductivity loss (45–48%) due to π−π stacking and H-bonding.	[33]
Mineral Scaling	Seawater Desalination	$Mg^{2+}$, $Ca^{2+}$, $OH^{-}$	“Scaling-enhanced scaling” via water splitting; effective area reduction.	[34]
Mineral Scaling	Laboratory ED	Gypsum ($CaSO_{4}$)	Internal scaling in heterogeneous membranes is irreversible.	[35]
Mineral Scaling	Electrochemical Descaling	$CaCO_{3}$	SEC: 1.2 kWh/kg (BPM) vs 7–10 kWh/kg (CEM).	[36]
Biofouling	RED/Bio-electrochemical	Biofilms (*P. aeruginosa*)	Biofilm-enhanced concentration polarization (BECP).	[37]
Biofouling	General ED/RO	Bacterial EPS	Major contributor (>45%) to total fouling; pressure drop increase.	[38]
Combined Fouling	Forward Osmosis	Alginate + Gypsum	Synergistic effect: organic layer traps crystals, accelerating flux decline.	[26]

## Data Availability

Dataset available on request from the authors.
